# Hydrological Response to Land Cover Changes and Human Activities in Arid Regions Using a Geographic Information System and Remote Sensing

**DOI:** 10.1371/journal.pone.0125805

**Published:** 2015-04-29

**Authors:** Shereif H. Mahmoud, A. A. Alazba

**Affiliations:** Alamoudi Water Research Chair, King Saud University, PO Box: 2460, 11451, Riyadh, Saudi Arabia; University of Sydney, AUSTRALIA

## Abstract

The hydrological response to land cover changes induced by human activities in arid regions has attracted increased research interest in recent decades. The study reported herein assessed the spatial and quantitative changes in surface runoff resulting from land cover change in the Al-Baha region of Saudi Arabia between 1990 and 2000 using an ArcGIS-surface runoff model and predicted land cover and surface runoff depth in 2030 using Markov chain analysis. Land cover maps for 1990 and 2000 were derived from satellite images using ArcGIS 10.1. The findings reveal a 26% decrease in forest and shrubland area, 28% increase in irrigated cropland, 1.5% increase in sparsely vegetated land and 0.5% increase in bare soil between 1990 and 2000. Overall, land cover changes resulted in a significant decrease in runoff depth values in most of the region. The decrease in surface runoff depth ranged from 25-106 mm/year in a 7020-km^2^ area, whereas the increase in such depth reached only 10 mm/year in a 243-km^2^ area. A maximum increase of 73 mm/year was seen in a limited area. The surface runoff depth decreased to the greatest extent in the central region of the study area due to the huge transition in land cover classes associated with the construction of 25 rainwater harvesting dams. The land cover prediction revealed a greater than twofold increase in irrigated cropland during the 2000-2030 period, whereas forest and shrubland are anticipated to occupy just 225 km^2^ of land area by 2030, a significant decrease from the 747 km^2^ they occupied in 2000. Overall, changes in land cover are predicted to result in an annual increase in irrigated cropland and dramatic decline in forest area in the study area over the next few decades. The increase in surface runoff depth is likely to have significant implications for irrigation activities.

## Introduction

Land cover change is defined as a change in land cover and its associated properties over time [1]. Such change is known to influence both surface water hydrology and soil hydraulic properties, as deforestation is likely to affect the local water balance [2], and the bulk density of soil increases with a change from forest to grassland or cropland [3]. Previous research has found land cover change to affect the hydrological response over a range of spatial scales [4]. It is known to affect both the balance between rainfall and evaporation and the runoff response by altering the physical structure of vegetation and surface roughness [2, 5]. Additionally, land cover change also alters the hydrological characteristics of the land surface and modifies the patterns and rates of water flow by determining the characteristics of runoff processes and the rates of infiltration, erosion and evapotranspiration (ET) [6].

The effects of land cover changes on the runoff of a catchment are of great interest to water resource planners, managers and decision-makers [7]. In addition, land use and land cover (LULC) changes, particularly those caused by human activities-for example deforestation to clear land for agriculture, are considered to be the most important factor in global environmental change, exerting effects possibly greater than those of other global changes [8, 9]. The mapping of land cover changes is essential for a wide range of applications, including those dealing with land planning and global warming [10]. Analysis of the potential effects of such changes on runoff requires a reliable database of both factors over a long period of time, a database that is seldom available, particularly in developing countries, which generally lack historical records on runoff or the hydrological effects of land cover change. Moreover, changes in climate factors may also influence runoff, and thus also need to be taken into account [1]. Changes in land cover are taking place at a rapid pace in the developing world, and deforestation activities are having a major impact on water resources [11], an issue of growing concern over the past few decades. However, analysis of the effects of land cover change on hydrological responses remains limited [12–14].

Research has shown that land cover classes can be used successfully to generate model inputs to assess the effects of land cover change on watershed responses [13], and several recent studies have estimated the effects of such change on hydrology in various parts of the world [15–19]. For example, one study [18] documented significant LULC change-induced stream flow changes and quantified the effects of those changes in the Laohahe Basin of China. LULC changes, dams, irrigation from rivers, industry, livestock and human consumption were all found to have significantly influenced the observed stream flow. A similar study [16] assessed the effects of land cover and climate change on the Gilgel Abay River of the Upper Blue Nile Basin in Ethiopia using the Soil and Water Assessment Tool, revealing both to have negligible effects on the river’s low-flow conditions.

Recent developments in decision support systems based on geographic information systems (GIS) and remote sensing (RS), and their combination with land use change models to assess the influence of land cover on biophysical processes, have led to practical tools for evaluating the hydrological responses of a watershed [2, 20–22]. RS data in the form of classified land cover data provide important information on land cover change and derive input variables for a wide variety of environmental models, including hydrological response, habitat, drought and land cover change models [16, 17, 23–29].

The Soil Conservation Service curve number (SCS-CN) method has been applied to a wide range of catchments across the world to assess the effects of land cover change on surface runoff [30–33]. The runoff coefficient can be defined as either the ratio of total runoff depth to total rainfall depth or the ratio of the peak rate of runoff to rainfall intensity for the time of concentration [27–29, 34, 35]. The runoff CN is widely used in hydrology to predict direct runoff or infiltration from excess rainfall. The rainfall-runoff relations within a watershed are driven primarily by the interplay of such factors as climate, land cover and soil [36–39]. Physically based distributed hydrological models have become a more feasible approach to flood prediction and rainfall-runoff computation in recent years [40]. Improved computational capabilities, in conjunction with digital elevation models (DEMs), digital data on soil type and land use, and GIS tools, are offering new possibilities for hydrological research, helping us to better understand the fundamental physical processes underlying the hydrological cycle and develop solutions to the mathematical equations representing those processes [40].

Markov chain analysis calculates a transition probability matrix of LULC changes from earlier to later dates, and then uses the transition as a basis to estimate future changes [41]. Numerous studies have found the integration of RS and GIS technologies with the Markov model and regression model to be beneficial to describing and analysing the land cover change process [41–53]. In this study, we used Markov chain model in IDRISI Selva software to model land cover changes and spatial distribution in the future. A Markovian process is one in which the state of a system at time *t*_2_ can be predicted by the state of the system at time *t*_1_ given a matrix of transition probabilities from each land cover class to every other cover class.

The effects of changing land cover patterns on water resources are creating social and political tensions at the local and national levels worldwide. In the Al-Baha region of Saudi Arabia, for example, the shift towards agriculture has generated a number of changes in the structure and function of the ecosystem, resulting in the overall degradation of the ecological services provided by region’s natural system. In addition, the forests of the Al-Baha Mountains have been largely destroyed, and they are further threatened by agricultural encroachment and the underplanting of forests with irrigated crops. Other disturbances include timber harvesting and livestock grazing. Although the amounts of discharge observed in the Al-Baha region appear to be lower than model predictions would suggest, no comprehensive discharge data are available for the region, and nor does it have any hydrological monitoring stations. Similarly, no data are available for the land cover changes that have been reported in the region. The study reported herein was carried out to address these gaps in our knowledge. Its objectives were as follows.

- To assess past and potential land cover changes and their effects on the hydrological response in the Al-Baha region of Saudi Arabia and to investigate historical climatic data (1950–2013).
- To determine the spatial and quantitative changes in surface runoff depth that resulted from land cover change between 1990 and 2000 using an ArcGIS-surface runoff model.
- To predict land cover and surface runoff depth in 2030 using Markov chain analysis.

### Study area

**Fig 1:**
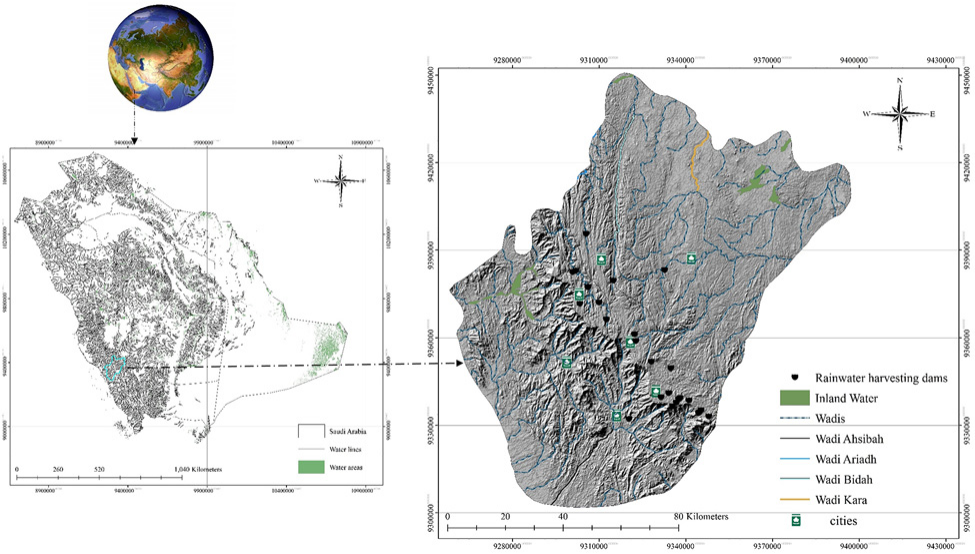
Location map of study area.

The Al-Baha region (Fig 1) is situated in Hejaz in the western part of the Kingdom of Saudi Arabia (41° 42′ E, 19° 20′ N). The smallest of the Kingdom’s provinces at 12,000 km^2^, Al-Baha was selected for this study because of the considerable divergence in its topography and climate. Al-Baha Province comprises six main districts, four of which are located in the Al-Sarah sector adjacent to ‘downtown’ Al-Baha, i.e. Al-Aqiq, Al-Mandaq, Al-Qura and Baljurashi, and two of which are in the Tihama sector, i.e. Al-Mekhwa (which includes Dhee Ain Village, or the Marble Village) and Qelwa. The climate of Al-Baha Province is greatly influenced by its varying topography. It is generally moderate in summer and cold in winter, with average temperatures ranging from 12–23°C. In Tihama, the climate is hot in summer, warm in spring and mild in winter, with humidity levels ranging between 52% and 67% and annual rainfall of less than 100 mm [30]. Although it is just 30 km from Tihama, the climate of the mountainous Al-Sarah area differs considerably. It is cooler in both summer and winter due to its high altitude. Al-Sarah is also subject to the formation of clouds and fog, which often occurs in winter because of the air masses coming in from the Red Sea and are accompanied by thunderstorms. In spring and summer, the climate is mild and pleasant. Al-Sarah also has higher annual rainfall than Tihama, in the range of 229–581 mm. Province-wide, average annual rainfall ranges from 100–250 mm [38].

## Materials and Methods

The study’s implementation required research techniques developed in a variety of fields. In addition to office work and field surveys, it involved the use of GIS, IDRISI Selva, land change modelling and Markov chain analysis. These techniques were used to identify past, and predict future, land cover changes and their effects on the hydrological response. The following materials and software were also used in the study’s implementation: the ESRI ArcGIS Spatial Analyst extension, ERDAS Imagine 2013 and satellite imagery. The main data collected were as follows

- Soil texture map
- DEM
- Ground truth point for land cover generation
- TM and ETM Landsat satellite imagery for the Al-Baha region (1990–2000)
- Climatic data (1950–2013)

**Fig 2:**
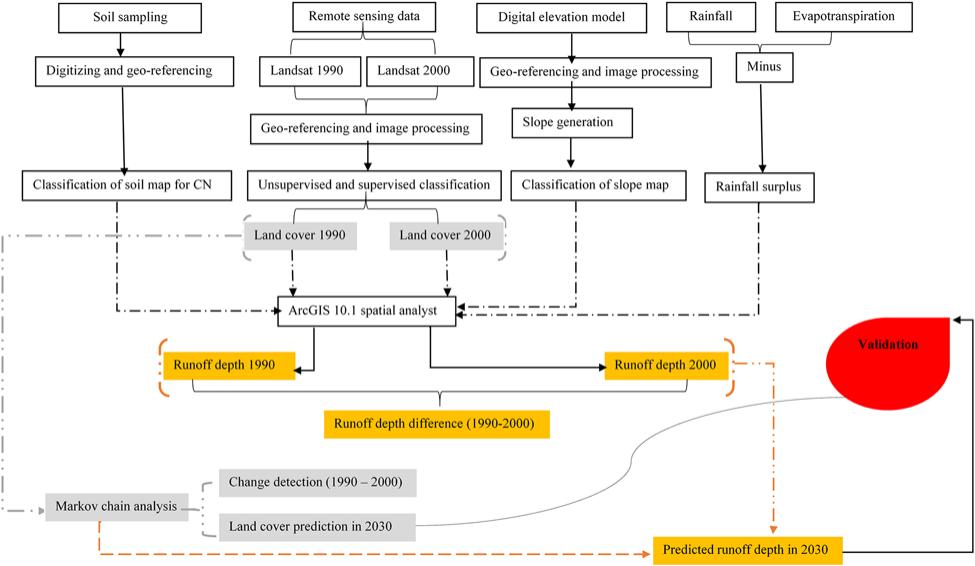
Workflow chart for estimating hydrological response to land cover changes.

Assessing the spatial changes in the annual average surface runoff depth between 1990 and 2000 and estimating the hydrological response to land cover changes and projections was carried out in several steps, as shown in the workflow chart in Fig 2. Field surveys were conducted between June and August in 2012 and 2013. As they were carried out in public locations, no permission was required for the field surveys. Fieldwork included geomorphological and land cover mapping of the selected periods, as well as GPS-based observations of soil textures, neither of which posed a threat to endangered or protected species. Land cover mapping and change detection analysis were performed by classifying cloud-free Landsat images from 1990 and 2000. Data were processed using ERDAS Imagine 2013, IDRISI Selva 17 and ArcGIS 10.1 software. In addition, we used a 2000 land cover map [38] for verification of the satellite image-based land cover classification. During the field surveys, visualisation of the specific land cover was made to collect ground truth points for classification and observe the human effects on land cover changes. More than 500 ground truth points were collected during the two field surveys. The land cover classification was based on these ground truth points using geocoded ground observation points and visual interpretations of Google Earth images.

### Soil texture map

The soil map for the study area developed in a previous study [30] using a GPS survey was used with the support of soil experts to identify the soil texture in the study sites. Soil samples were collected from the entire study area and then analysed in ArcGIS to develop a soil texture map for the study area. The map was divided into three classes of soil: loam, clay and silty clay. The results revealed 85.8% of the study area to be made up of loamy soil, which has a moderate infiltration rate when it is thoroughly saturated, and is classified as having mainly or moderately deep infiltration and moderately to well-drained soil with moderately fine to moderately coarse textures. In addition, 8.4% of the area was classified as silty clay, which has low infiltration rates, and 5.7% as clay soil, which has the lowest infiltration rate. Two hydrological soil groups in the study area were also found to have the following characteristics. First, Group B comprised silty loam or loam soils, which have a moderate infiltration rate when wet and are classified as having mainly or moderately deep infiltration and moderately to well-drained soil with moderately fine to moderately coarse textures. Second, Group D comprised clay loam, silty clay loam, sandy clay, silty clay and clay soils. These soils have the greatest runoff potential. They have very low infiltration rates when thoroughly wet and consist chiefly of clay soils with high swelling potential, soils with a permanent high water table, soils with a clay pan or clay layer at or near the surface, and shallow soils over nearly impervious material.

### Land cover classification

#### Data pre-processing

Landsat surface reflectance 5/7 TM/ETM cloud-free images for 1990 and 2000 were obtained from King Abdulaziz City for Science and Technology (KACST) (Table 1). These images were geometrically corrected and projected to the World Geodetic System (WGS 84—UTM zone 37N) using an already rectified SPOT-5 “2.5m PSM” satellite image of 06/03/2000. This image was also employed as reference data and a base map for the assessment of classification and accuracy. The Landsat images were resampled using the nearest neighbour algorithm to keep the original brightness and pixels values. The resultant root mean squared error of each image was less than 0.54 pixel (1.35 m). The Cost model in IDRISI Selva was used for the atmospheric correction of all the Landsat and SPOT images. Using the technique of image normalization, Landsat images were then indirectly normalized for atmospheric absorption with the SPOT-5 image being the reference image. The two Landsat images have been radiometrically normalised to a base image (SPOT-5 “2.5m”) using manually identified pseudo-invariant features (PIFs). After identifying 12 PIFs, a linear regression was used to place the data at each band onto the same radiometric reference.

**Table 1:** Remote sensing data used for the study.

Images Used for the study	Resolution (m)	Date of acquisition	Format	Product Type (Cloud Cover %)
Landsat 5 TM “Band 1–7”	30	24/02/1990	surface reflectance	L1T* (0%)
Landsat 7 ETM+ “Band 1–8”	30	4/1/2000	surface reflectance	L1T (0%)
SPOT-5 “PSM”	2.5	6/3/2000	surface reflectance	L1T (0%)

*Level 1T (L1T) (precision and terrain corrected data) provides systematic radiometric accuracy, geometric accuracy by incorporating ground control points, while also employing a Digital Elevation Model (DEM) for topographic accuracy [80].

#### Data processing and land cover classification

After pre-processing, the Landsat images were then processed using ERDAS Imagine 2013, IDRISI Selva 17 and ArcGIS 10.1 softwares. In addition, we used a 2000 land cover map and other ground values’ data [38] for verification of the satellite image-based land cover classification. During the field surveys, visualisation of the specific land cover was made to collect ground truth points for classification and observe the human effects on land cover changes. More than 500 ground truth points were collected during the two field surveys (S1 Fig). The land cover classification was based on these ground truth points using geocoded ground observation points and visual interpretations of Google Earth images.

Images classification was performed using the Iso Cluster Unsupervised Classification tool in ArcGIS 10.1 Spatial Analyst to define the signature files and fix the number of classes. The resulting raster layer provided delineation of the land cover classes in the satellite images. Classes with a similar value were merged. Unsupervised classification revealed four land cover classes, which were then verified by training samples collected during the field surveys to create spectral signatures (i.e. reflectance values). Using the maximum likelihood classification method and the previous collected ground data, we identified what each cluster represented (e.g. water, bare earth, dry soil, etc.). Furthermore, the land cover in the maps was classified into four main classes: bare soil, sparsely vegetated land, forest and shrubland, and irrigated cropland. Finally, the land cover classification output was subjected to accuracy assessment.

### Modelling land cover changes and prediction

Change detection is the process of identifying differences in LULC amongst multiple RS data for an area. Various techniques are used in conjunction with satellite imagery in change detection, including principal component analysis, image differencing, spectral vector analysis and post-classification [9, 54]. In this study, land cover change and dynamics were determined using the Land Change Modeler (LCM) in IDRISI 17 (Selva Edition). The results were evaluated using a change detection matrix, with detailed ‘from-to’ information then extracted from the matrix. The area percentages of three classified images were first plotted by category to observe the trends in land cover change. Then, the earlier and later classified images in the LCM change analysis panel were used. This panel provided gain and loss graphs and net change graphs for each temporal period by category for calculation of the area and area percentage of changes. Next, the additional ‘from-to’ information was computed using the crosstab module, which provided a cross-classification image showing the locations of the categories in the earlier image that were the same as those in the second image, and vice versa. Finally, the earlier classified images and cross-classification images for each period were used to determine the relative frequency of the different land cover categories occurring in areas of transition. Transition probability and the quantity of future changes were modelled through Markov chain analysis. The output from the transition prediction was the land cover transition probability in 2030.

### Digital elevation model

A 30-m DEM 30 obtained from KACST was used to generate the slope map for the Al-Baha region. DEM analysis was performed to remove sinks and flat areas to maintain flow continuity to the catchment outlets. A GIS was used to fill the sink areas to prepare the DEM for the next step. A slope degree map was then generated for the study area from the Al-Baha filled DEM. The slope map created for the entire area gives an impression of the steepness of the terrain.

### Meteorological data

Climatic data were collocated for a period of 63 years to obtain long-term annual rainfall data for the study area. These data were also used to develop an ET model in ArcGIS 10.1 ModelBuilder, which was subsequently used to calculate the potential average annual ET using the Penman-Monteith method and to identify the average annual rainfall surplus using long-term annual rainfall and average annual ET data. The climatic data obtained from the Meteorological Department of the Ministry of Agriculture and the Ministry of Water and Electricity were insufficient, and thus additional data were interpolated using the following sources.

- Satellite images for monthly global precipitation from 1979 to 2009 were obtained from the World Data Center for Meteorology.
- Monthly global precipitation data from the NASA Tropical Rainfall Measuring Mission Monthly for the 1998–2010 period were obtained from NASA’s Goddard Earth Sciences Distributed Active Archive Center.

The Penman-Monteith method [55] was used to estimate the potential ET:

$$E_{T}=\frac{\Delta R_{n}+(e_{a}-e_{d})*\frac{\rho *c_{p}}{r_{a}}}{\lambda (\Delta +\gamma *(1+\frac{r_{s}}{r_{a}}))}$$ (1)

where

R_n_ = net radiation (W/m^2^);

ρ = density of air;

c_p_ = specific heat of air;

r_s_ = net resistance to diffusion through surfaces of leaves and soil (s/m);

r_a_ = net resistance to diffusion through the air from surfaces to the height of the measuring instruments (s/m);

γ = hygrometric constant;

Δ = de/dT;

e_a_ = saturated vapour pressure at air temperature; and

e_d_ = mean vapour pressure.

ET refers to the total amount of water vapour entering the atmosphere through either the evaporation of water from the surfaces of open water and soil or its transpiration from the leaves of vegetation. ET estimation constituted a long-standing major scientific challenge until the development of a combination approach suitable for open water and wet soil surfaces [56] and an improved model for the unsaturated surface of a single leaf that introduced resistance [57]. The Penman equation was initially applied to the vegetative canopy [55], and later evolved into the well-known Penman-Monteith equation. The amount of ET is equally expressed in two units: the amount of water that has left the surface via ET (mm) and the amount of energy used in ET (W/m^2^). A rainfall surplus (P-ET) map was calculated by subtracting the long-term average monthly ET values of precipitation for all meteorological stations covering the 1950–2013 period. The annual rainfall surplus was calculated at each meteorological station by adding only the positive values of the difference (P-ET), and the spatial distribution of the rainfall surplus was generated by interpolating previous data values using ArcGIS.

### Surface runoff generation

Various models are used to investigate the watershed response to land cover and climate changes and the effects of climatic variability on the hydrological process, with minor modifications for different applications [11, 58–61]. The rainfall runoff relations in the study area were evaluated using the SCS-CN method, which is suitable because of its reliance on land cover parameters. The SCS-CN method has several advantages, chief amongst which is its ease of use and widespread acceptability, but it is also has several disadvantages. Nevertheless, its use is considered appropriate in the absence of accurate hydrological and topographical data for runoff estimation [62]. The method’s stability is ensured by the surface runoff depth (Q) being bounded between 0 and the maximum rainfall depth (P), which implies that, as the amount of rainfall increases, the actual retention (P-Q) approaches a constant value, i.e. the maximum potential retention [63, 64]. The runoff coefficient can be derived as either an event runoff coefficient or an annual runoff coefficient, with the former defined as the portion of rainfall that becomes direct runoff during a particular event. In hydrological modelling, it represents the lumped effect of a number of processes in a catchment, which may include interception, evaporation, rainfall intensity, initial abstraction and, hence, runoff [65].

**Fig 3:**
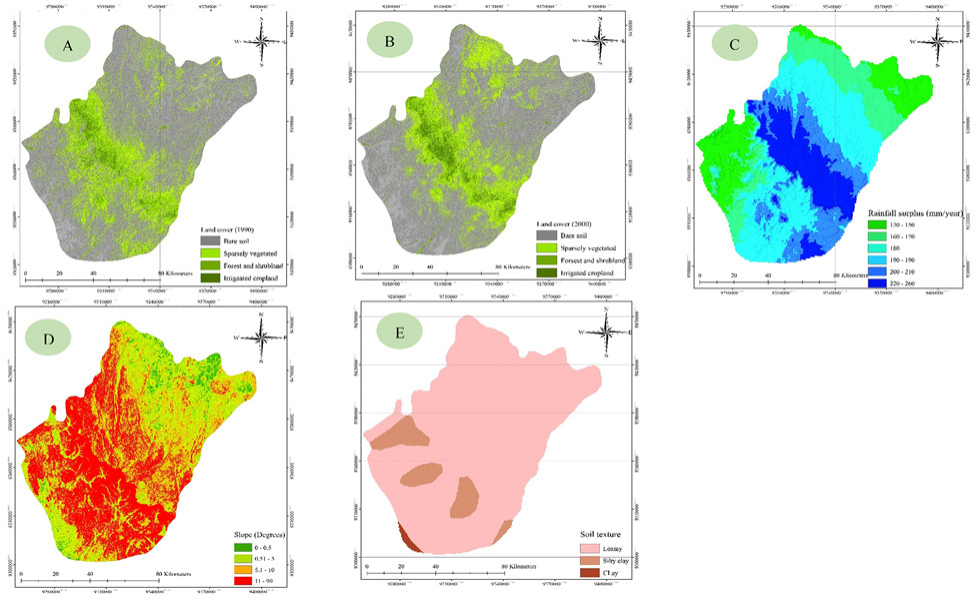
Factors considered in the determination of surface runoff depth: (A) land cover 1990, (B) land cover 2000, (C) rainfall surplus, (D) slope and (E) soil texture.

Land cover data are required to achieve baseline climate data, and can also be used to assess the effects of land cover changes and other climate variables in an area’s hydrological response. In the study reported herein, the factors considered in determining the surface runoff depth (see Fig 3) were (1) the land cover map in 1990, (2) land cover map in 2000, (3) rainfall surplus (long-term annual average), (4) slope degree map and (5) soil texture. These factors were also used to develop an ArcGIS surface runoff model for the two periods. This model presents a hydrological parameter that can be used to describe the potential surface runoff depth for drainage areas, and is a function of land cover, soil type, slope and rainfall surplus. CN values were first imported into the model as an Excel file from the potential runoff coefficient tables (Tables 2 and 3). These tables were developed for runoff values based on soil type, land cover and slope degree in previous research [66], and discretise surface slope into four classes based on those values. The values in the two tables are taken from the literature [67–70]. Finally, the annual surface runoff depth was derived for the two periods rather than obtaining an event runoff coefficient for the annual rainfall surplus.

**Table 2:** Potential runoff coefficient for different land use, soil type and slope.

Land use	Slope	Sand	Loamy	Sandy	Loam	Silt	Silt	Sandy	Clay	Silty	Sandy	Silty	Clay
								clay		clay			
	(%)		sand	loam		loam		loam	loam	loam	clay	Clay	
Forest	<0,5	0.03	0.07	0.10	0.13	0.17	0.20	0.23	0.27	0.30	0.33	0.37	0.40
	0,5–5	0.07	0.11	0.14	0.17	0.21	0.24	0.27	0.31	0.34	0.37	0.41	0.44
	5–10	0.13	0.17	0.20	0.23	0.27	0.30	0.33	0.37	0.40	0.43	0.47	0.50
	>10	0.25	0.29	0.32	0.35	0.39	0.42	0.45	0.49	0.52	0.55	0.59	0.62
Grass	<0,5	0.13	0.17	0.20	0.23	0.27	0.30	0.33	0.37	0.40	0.43	0.47	0.50
	0,5–5	0.17	0.21	0.24	0.27	0.31	0.34	0.37	0.41	0.44	0.47	0.51	0.54
	5–10	0.23	0.27	0.30	0.33	0.37	0.40	0.43	0.47	0.50	0.53	0.57	0.60
	>10	0.35	0.39	0.42	0.45	0.49	0.52	0.55	0.59	0.62	0.65	0.69	0.72
Crop	<0,5	0.23	0.27	0.30	0.33	0.37	0.40	0.43	0.47	0.50	0.53	0.57	0.60
	0,5–5	0.27	0.31	0.34	0.37	0.41	0.44	0.47	0.51	0.54	0.57	0.61	0.64
	5–10	0.33	0.37	0.40	0.43	0.47	0.50	0.53	0.57	0.60	0.63	0.67	0.70
	>10	0.45	0.49	0.52	0.55	0.59	0.62	0.65	0.69	0.72	0.75	0.79	0.82
Bare	<0,5	0.33	0.37	0.40	0.43	0.47	0.50	0.53	0.57	0.60	0.63	0.67	0.70
soil	0,5–5	0.37	0.41	0.44	0.47	0.51	0.54	0.57	0.61	0.64	0.67	0.71	0.74
	5–10	0.43	0.47	0.50	0.53	0.57	0.60	0.63	0.67	0.70	0.73	0.77	0.80
	>10	0.55	0.59	0.62	0.65	0.69	0.72	0.75	0.79	0.82	0.85	0.89	0.92
IMP		1.00	1.00	1.00	1.00	1.00	1.00	1.00	1.00	1.00	1.00	1.00	1.00

**Table 3:** Slope constant S_0_ for determining potential runoff coefficient.

							Sandy		Silty			
Land use	Sand	Loamy	Sandy	Loam	Silt	Silt	clay	Clay	clay	Sandy	Silty	Clay
	sand		loam		loam		loam	loam	loam	clay	clay	
Forest	0.68	0.65	0.62	0.59	0.56	0.53	0.5	0.47	0.44	0.41	0.38	0.35
Grass	0.58	0.551	0.522	0.493	0.464	0.435	0.405	0.376	0.347	0.318	0.289	0.26
Crop	0.5	0.471	0.442	0.413	0.384	0.355	0.325	0.296	0.267	0.238	0.209	0.18
Bare soil	0.42	0.393	0.365	0.338	0.311	0.284	0.256	0.229	0.202	0.175	0.147	0.12

### Surface runoff differences and prediction

To determine the spatial and quantitative changes in surface runoff depth resulting from land cover change between 1990 and 2000, the annual surface runoff depth in 2000 was subtracted from that in 1990. The output was a new raster image representing the changes in the annual such depth. The same methodology as that used for surface runoff generation was used to spatially model the values of the predicted annual surface runoff depth in 2030 using Markov chain analysis. The predicted runoff depth was based on the change in modelled runoff depth between 1990 and 2000. Finally, the surface runoff model was used to validate the predicted runoff depth for 2030 based on hydrological soil group, slope degree, 2030 land cover map and rainfall surplus.

## Results and Discussion

The hydrological response to human activity-induced land cover changes in arid regions has attracted increased research attention in the past few years due to the resulting effects on water resources and the environment. In this study, the research period and area were selected on the basis of environmental concerns resulting from the establishment of dams and a decrease in forest area.

### Detection of land cover change

Monitoring of the locations and distributions of land cover changes constitutes an important step towards establishing links between the policy decisions, regulatory actions and subsequent land-use activities that will lead to sustainable management of the environment and highlight the risks for future generations. In this study, land cover maps for 1990 (Fig 3A) and 2000 (Fig 3B) were derived from cloud-free satellite images using ERDAS Imagine 13 software and ArcGIS 10.1 Spatial Analyst. These maps were then verified using a 2000 land cover map obtained from published sources.

Accuracy assessment is an important element of image classification, and can be determined by an error matrix. Error matrices quantitatively compare the relation between classified images and reference data, which can include field observations, high-resolution satellite images and/or thematic maps. After an error matrix has been generated, overall accuracy and Kappa statistics can be developed [9, 71–75, 79]. In this study, the classification accuracy of the classified images was determined by both simple random and stratified random patterns. The simple random pattern provides an equivalent probability of sampling over the entire study area with no operator bias. To achieve it, 120 reference pixels are used for accuracy assessment, resulting in 98% accuracy with an acceptable error of 5% [76]. The resampling function in IDRISI Selva was used to improve the resolution and accuracy of the images in this study.

**Fig 4:**
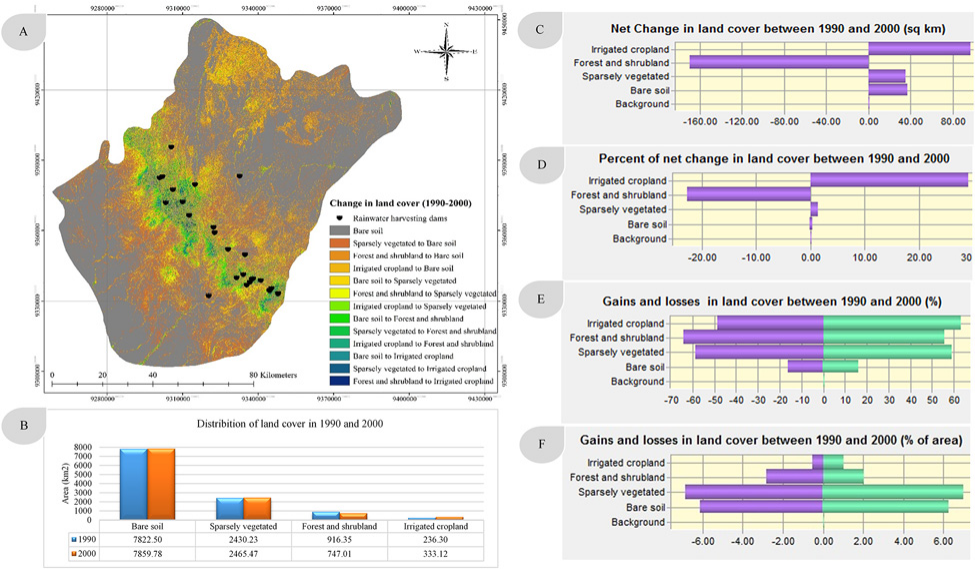
Spatial distribution of surface runoff depth in 1990 and 2000.

Combining image enhancement and post-classification analysis reduces change detection errors and provides detailed from-to change information [77, 78]. The land cover change map (from-to) in this study was produced using this combined approach (Fig 4A), and revealed extensive growth in irrigated cropland at the expense of forest, shrubland and grassland, and bare soil. A significant monotonic trend and marked changes in annual temperature have occurred over time.

The classified images revealed four main classes: bare soil, sparsely vegetated land, forest and shrubland, and irrigated cropland. The images suggest that land cover changes have had a negligible effect on forest and shrubland, which may have affected the ecosystem and natural balance of the study area. The results of land cover change analysis identified the expansion of irrigated cropland in 2000. The extent of the land cover distribution in 1990 and 2000 is presented in Fig 4B and Table 4. A slight increase in bare soil was observed, rising from 7822 km^2^ in 1990 to 7859 km^2^ in 2000, and a similarly slight increase in sparsely vegetated land, which rose from 2430 km^2^ to 2465 km^2^ in 2000. However, forest and shrubland declined during the period, falling from 916 km^2^ in 1990 to 747 km^2^ in 2000, which also witnessed an increase in irrigated cropland relative to 1990 (333 km^2^ versus 236 km^2^). The land cover change in the study area between 1990 and 2000 is shown in percentage terms in Fig 3C and 3D. In addition, Fig 4E and 4F show the net change as a percentage of each class and of the total area, respectively. These figures show a 26% decrease in forest and shrubland, a 28% increase in irrigated cropland, a 1.5% increase in sparsely vegetated land and a 0.5% increase in bare soil between 1990 and 2000.

**Table 4:** The extent of land cover distribution in 1990, 2000 and the predicted cover in 2030.

	1990	2000	2030
Land cover class	Area (Km^2^)	Area (Km^2^)	Area (Km^2^)
Bare soil	7822.50	7859.78	7967.90
Sparsely vegetated	2430.23	2465.47	2567.66
Forest and shrub land	916.35	747.01	255.92
Irrigated cropland	236.30	333.12	613.91

### Hydrological response to land cover change

**Fig 5:**
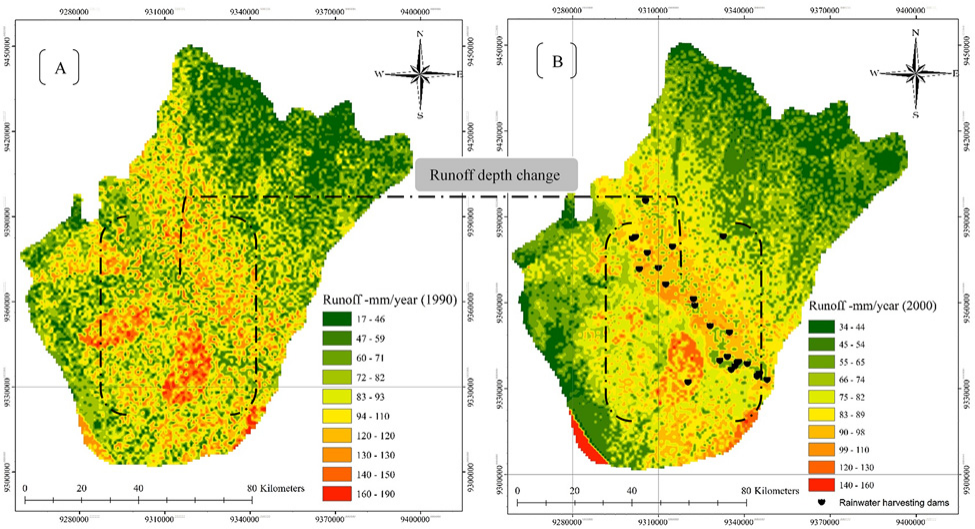
Changes in annual surface runoff depth between 1990 and 2000; (-) indicates an increase in surface runoff values from 1990 and (+) a decrease in runoff depth values in 2000.

The results of the spatial distributions of modelled annual runoff depth (in mm) in 1990 and 2000 are shown in Fig 5A and 5B, respectively. The surface runoff depth in 1990 was much higher than that in 2000, when it varied from 17 to 190 mm/year, particularly in the mountainous areas with forest cover and shrubland. The significant decline in surface runoff can be attributed to the conversion of forest and shrubland to irrigated cropland. Another reason is the construction of 25 rainwater harvesting and recharge dams in the area, which control surface runoff and allow farmers to extend their fields and increase their income at the expense of forest and shrubland.

In 2000, rainfall varied from as little as 34 mm to as much as 162 mm due to the divergence in topography and climate in the study area caused by human activities and the construction of new water projects, although there was an annual increase in rainfall over the area as a whole, particularly in the mountainous areas. However, there was also a significant increase in the annual temperature because of deforestation over most of the region. As noted during the field surveys, the mountainous area in the Al-Baha region is subject to the formation of clouds and fog, particularly in winter when air masses come in from the Red Sea. The mountainous area also had the largest runoff depth of the various study sites due to its soil type and land cover conditions and the steepness of its slope. Moreover, the annual runoff depth exhibited wider variation over the study area in 1990 than in 2000.

**Fig 6:**
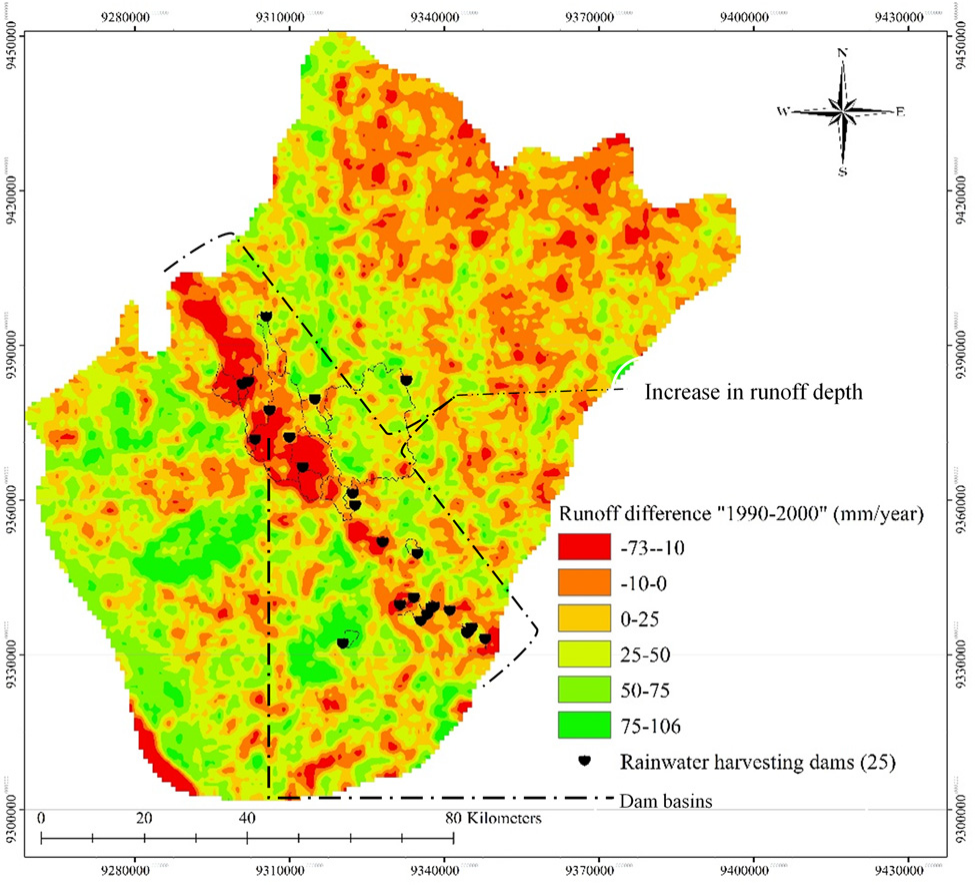
Average change in land cover between 1990 and 2000 in the study area.

Overall, land cover changes resulted in a significant decrease in surface runoff depth (Fig 6). That decrease (Table 5) ranged from 25–106 mm/year in an area totalling 7020 km^2^, whereas the increase in surface runoff depth amounted to just 10 mm/year in a 243 km^2^ area, with a maximum increase of up to 73 mm/year seen in a limited area. The surface runoff depth decreased to the greatest extent in the central region of the study area due to the huge transition in land cover classes associated with the construction of 25 rainwater harvesting dams. The predicted change in land cover revealed a greater than twofold increase in irrigated cropland during the 2000–2030 period, whereas forest and shrubland are anticipated to occupy just 225 km^2^ of land area by 2030, relative to 747 km^2^ in 2000.

**Table 5:** The extent of the areas where surface runoff depth changes occurred with the study area (1990–2000).

Runoff difference (1990–2000) mm	Area Km^2^
-73	0.81
-10	2473.14
0	1723.21
25	3447.23
50	3001.31
75	512.04
106	60.48

### Hydrological effects of human activities

Twenty-five dams were found within the study area during the field surveys, 17 of which had been established to recharge groundwater supplies. Construction of these dams was justified because the area’s groundwater resources had been depleted before it commenced. Such depletion over the years has hindered the area’s agricultural activities because groundwater wells are the main source of water. The surveys revealed only one dam is used for irrigation purposes and other activities, and three dams for flood control. In addition, four dams were established for drinking purposes in areas in which desalinated water is somewhat difficult to obtain. In general, the main purpose of the existing dams in the study area is to recharge groundwater supplies in support of the agricultural sector, which highlights the significance of this study.

The change in flow path resulting from the construction of rainwater harvesting dams affected the land cover in the study area during the research period. In 1990, there was limited human interaction with the environment, and forest cover dominated the area. However, conditions changed significantly thereafter, and following the dams’ construction no water flowed to the streams or forestland, causing significant damage to wild plants, shrubland and forest cover. Overall, the effects of land cover change on the hydrology of the study area can be summarised as follows: such change (1) affected the area’s ecosystem, (2) resulted in considerable deforestation due to a dam-induced absence of water and (3) decreased surface runoff values.

### Prediction of land cover and surface runoff

**Fig 7:**
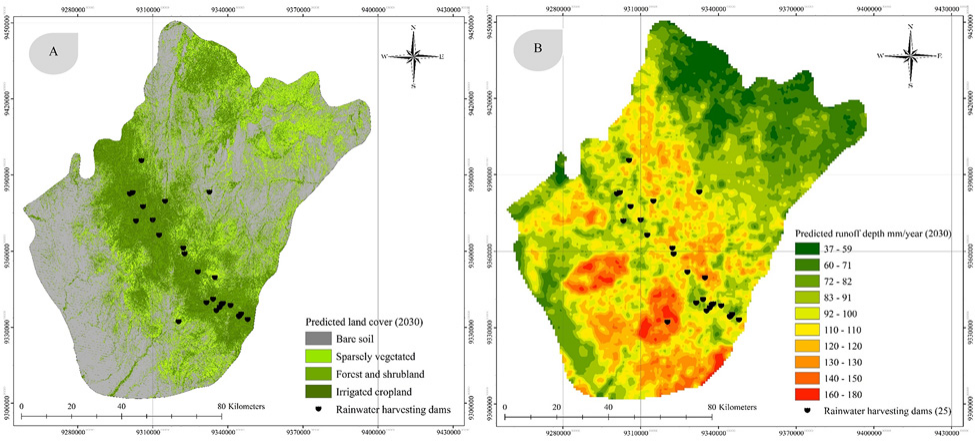
Predicted land cover in 2030 (A) and spatial distribution of the predicted surface runoff depth in 2030 (B).

Land cover change has significant effects on the functioning of socioeconomic and environmental systems and important benefits for sustainability. However, predicting the future effects of land cover and climate changes on an area requires an understanding of the effects that historic land cover changes have exerted on the hydrological regime. In this study, land cover change was projected for the next 16 years using a Markov chain model and regression analyses. The projected land cover in 2030 (Fig 7A) shows a continued increase in irrigated cropland at the expense of forest and shrubland, which has significant environmental implications for the study area. The anticipated land cover distribution in 2030 is presented in Table 3 and Fig 7A. The projections show a slight increase in bare soil, from 7859 km^2^ in 2000 to 7967 km^2^ in 2030, and a similarly slight increase in sparsely vegetated land, which is predicted to rise from 2465 km^2^ in 2000 to 2567 km^2^ in 2030. In contrast, forest and shrubland are likely to face a significant decline by 2030, decreasing to 255 km^2^ from 747 km^2^ in 2000. Irrigated cropland is also slated to expand dramatically by 2030, being predicted to occupy an area of 613 km^2^ instead of the 333 km^2^ area it occupied in 2000, a rise of 100%.

Annual surface runoff depth in 2030 (Fig 7B) is likely to increase in the mountainous areas (the central region of the study area), providing ideal conditions for rainwater storage and improved agriculture. Overall, changes in land cover will result in an annual increase in irrigated cropland and a dramatic decline in forest cover in the study area. The increase in surface runoff depth is likely to have significant implications for irrigation activities. Moreover, it will also help to harness a significant amount of rainwater in the existing rainwater harvesting dams in the Al-Baha region.

## Conclusion

The study reported herein assessed the spatial and quantitative changes in surface runoff depth that resulted from land cover change between 1990 and 2000 in the Al-Baha region of Saudi Arabia using an ArcGIS-surface runoff model. This model presents a hydrological parameter that is used to describe the surface runoff depth for drainage areas, and is a function of land cover, soil type, slope and rainfall surplus. In addition, the study predicted land cover and surface runoff depth in 2030 using Markov chain analysis. Analysis of land cover changes detected on processed satellite images revealed progressive such changes in the Al-Baha region between 1990 and 2000. The 25% decline in forest cover and 28% increase in irrigated cropland over the 10-year period are both attributable to the agricultural sector. Similarly, a substantial amount of sparsely vegetated land was also converted to agricultural use during the study period.

This agricultural expansion occurred as a result of rapid increases in population and human activity and the establishment of large-scale water projects such as dams and flood control infrastructure. Moreover, poor resource management resulted in the overexploitation of natural resources, with adverse effects on sustainable development. Land cover conversion has resulted in land degradation, interfered with biodiversity and the ecosystem, and caused water stress in forest areas, thus depriving the region’s wild animals of a much-needed source of water. Consequently, many wild animals have migrated to cities and villages in search of water and are fighting for their very survival.

Land cover is known to exert major effects on a rage of hydrological processes, including runoff, ET and groundwater flow. In this study, the decrease in surface runoff depth ranged from 25–106 mm/year in an area totalling 7020 km^2^, whereas the increase in such depth reached just 10 mm/year in a 243 km^2^ area, with a maximum increase of 73 mm/year in a limited area. The surface runoff depth decreased to the greatest extent in the central region of the study area due to the huge transition in land cover classes associated with the construction of 25 rainwater harvesting dams. The land cover prediction revealed a more than twofold increase in irrigated cropland during the 2000–2030 period, whereas forest and shrubland are anticipated to occupy just 225 km^2^ of land area in 2030, a significant decrease from the 747 km^2^ they occupied in 2000.

Changes in rainfall and temperature were used in the surface runoff model. The predicted rise in the region’s annual average temperature in the coming years is expected to be accompanied by an increase in rainfall, particularly in mountainous areas, although it will vary depending on the extent of deforestation. In addition, ET will tend to rise in the central region in conjunction with the anticipated expansion of agricultural activities. The projected land cover in 2030 shows a continued increase in irrigated cropland at the expense of forest and shrubland, which has significant environmental implications for the study area. However, the annual surface runoff depth in the mountainous areas (the central region) may be higher in 2030, thereby providing ideal rainwater storage conditions and improving the prospects of the Al-Baha region’s agricultural sector.

## Supporting Information

S1 Fig Field studies. (TIF)
