# Supplementary figures and images for: Hydrological Response to Land Cover Changes and Human Activities in Arid Regions Using a Geographic Information System and Remote Sensing
Source: PLoS One. 2015 Apr 29;10(4):e0125805. doi: 10.1371/journal.pone.0125805 (PMC4414520; doi:10.1371/journal.pone.0125805)

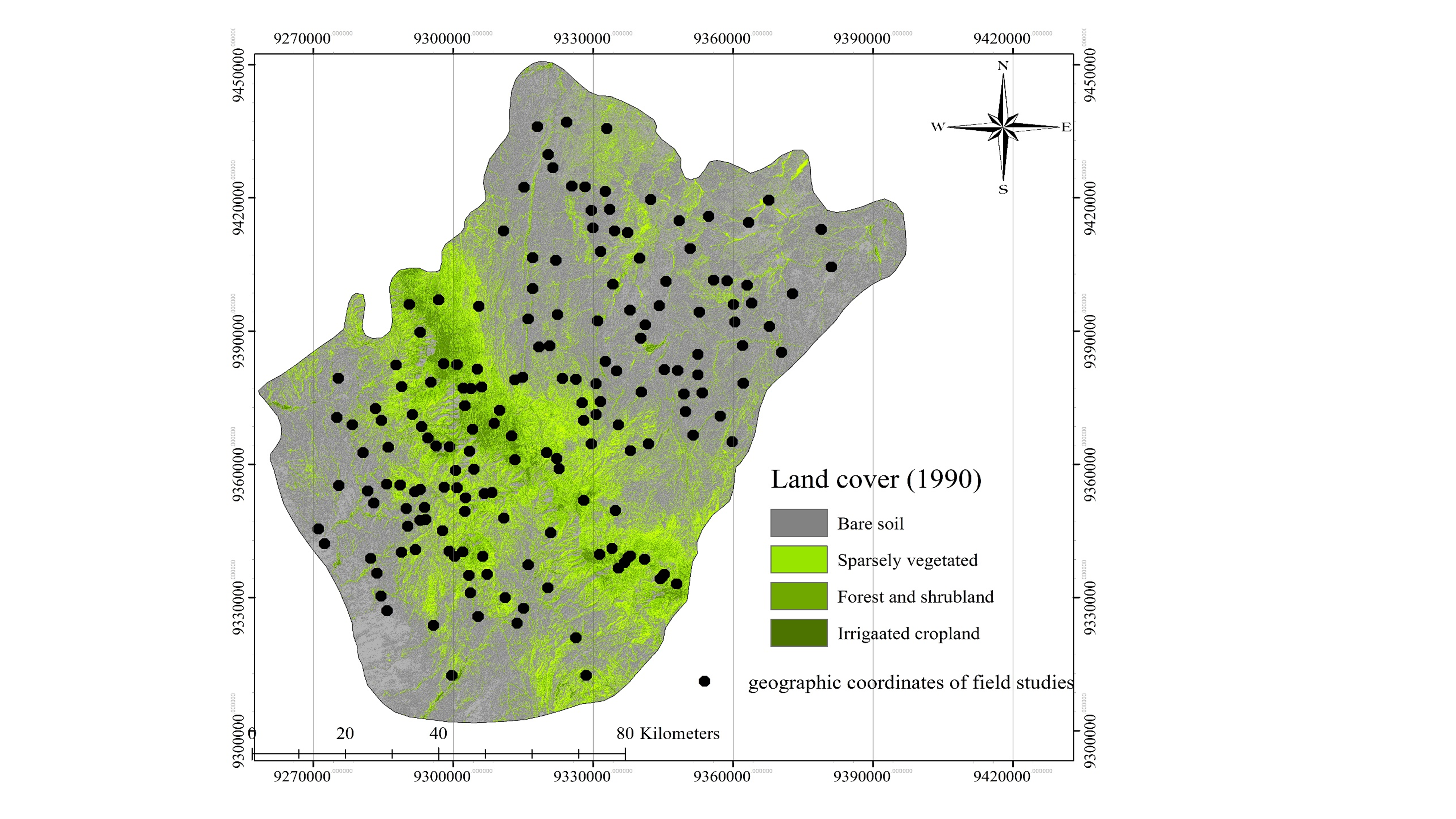

Supplement: S1 Fig — (TIF) [file pone.0125805.s001.tif]
